# Comparison of long-term outcome in patients with calcified stenosis treated with intravascular lithotripsy or with modified balloon angioplasty: a propensity score-adjusted study

**DOI:** 10.3389/fcvm.2023.1185422

**Published:** 2023-05-15

**Authors:** Jürgen Leick, Tobias Rheude, Michael Denne, Salvatore Cassese, Adnan Kastrati, Felix Hauptmann, Thomas Gehrig, Constantin Kuna, Michael Lindner, Michael Lauterbach, Nikos Werner

**Affiliations:** ^1^ Department of Cardiology, Heart Centre Trier, Barmherzige Brueder Hospital, Trier, Germany; ^2^ Department of Cardiovascular Diseases, German Heart Centre, Technical University Munich, Munich, Germany

**Keywords:** intravascular lithotripsy, modified balloon, calcified coronary arteries, lesion preparation, stent implantation

## Abstract

**Background:**

The aim of this two-center, all-comers registry was to compare the effectiveness and safety of intravascular lithotripsy (IVL) to that of modified balloon angioplasty (MB). MB angioplasty using a cutting or scoring balloon is commonly used in patients with calcified coronary arteries. IVL is a new technology for lesion preparation. This is the first study to compare MB with IVL.

**Methods:**

The cohort included all patients treated by MB angioplasty or IVL between 2019 and 2021. The primary endpoint was strategy success (<20% residual stenosis). The secondary endpoint was long-term safety outcomes [cardiac death, acute myocardial infarction (AMI), target lesion failure/revascularization (TVR)]. Quantitative coronary angiography (QCA) was performed in all patients. Primary and secondary endpoints were compared using inverse probability of treatment weighting (IPTW) for treatment effect estimation.

**Results:**

A total of *n* = 86 patients were treated by IVL and *n* = 92 patients by MB angioplasty. The primary endpoint was reached in 152 patients (85.4%). Patients in the IVL group had less residual stenosis (5.8% vs. 22.8%; *p* = 0.001) in QCA. Weighted multivariable regression analysis revealed that IVL had a significant positive effect on reaching the primary endpoint of strategy success [odds ratio (OR) 24.58; 95% confidence interval (95% CI) 7.40–101.86; *p* = 0.001]. In addition, severe calcification was shown to result in a lower probability of achieving the primary endpoint (OR 0.08; 95% CI 0.02–0.24; *p* = 0.001). During the follow-up period (450 days) there was no difference in cardiovascular mortality rate [IVL (*n* = 5) 2.8% vs. MB (*n* = 3) 1.7%; *p* = 0.129]. Patients with unstable angina at the time of the index procedure had the highest probability of cardiovascular death [hazard ratio (HR) 7.136; 95% CI 1.248–40.802; *p* = 0.027]. No differences were found in long-term rates of AMI (IVL 1.7% vs. MB 2.8%; *p* = 0.399; IVL HR 2.73; 95% CI 0.4–17.0; *p* = 0.281) or TVR (IVL 5.6% vs. MB 9%; *p* = 0.186; IVL HR 0.78; 95% CI 0.277–2.166; *p* = 0.626).

**Conclusion:**

IVL leads to a significantly better angiographic intervention outcome compared to MB angioplasty in our cohort. During long-term follow-up, no differences in cardiovascular mortality, rate of acute myocardial infarction, or target lesion failure/revascularization were observed.

## Introduction

Treatment of complex coronary artery stenosis, especially in severely calcified coronary vessels, remains an interventional challenge. Coronary artery calcification impairs the vascular compliance, leads to an abnormal vasomotor response, and has negative effects on myocardial perfusion (1). The presence of calcification in patients undergoing percutaneous coronary intervention (PCI) is associated with a worse outcome (1–3). Severe calcification is an independent predictor of delayed healing after newer-generation drug-eluting stent (DES) implantation and of stent underexpansion (4). Stent underexpansion is one of the most powerful predictors of stent thrombosis and/or in-stent restenosis (ISR) (5–7). Due to changing demographics, the prevalence of patients with severely calcified lesions is constantly increasing (1); thus, the demand for easy-to-use, balloon-based techniques for plaque modification in calcified coronary vessels is increasing.

Lesion preparation using a modified balloon (MB; cutting or scoring balloon) is commonly used for plaque modification. MB create a discrete longitudinal incision in the atherosclerotic target coronary segment during balloon inflation. MB may reduce the force needed to dilate an obstructive lesion compared with standard percutaneous transluminal coronary angioplasty balloons or noncompliant balloon catheters (8, 9). Nevertheless, several trials have failed to demonstrate an advantage of MB over conventional balloon angioplasty, non-compliant, or super high-pressure balloon dilatation (8, 10, 11). However, the data from the COPS study show that the use of a cutting balloon results in a larger minimal stent area than non-compliant balloons (12).

Intravascular lithotripsy (IVL) is a novel and safe technique for plaque modification (5, 13–15). In this recently approved technique, multiple lithotripsy emitters are integrated into a balloon system. These emitters generate a circumferential sound wave that leads to calcium fracturing and thus positively influences vascular compliance. Multicenter, non-randomized studies have demonstrated high rates of device and procedural success as well as good angiographic and clinical outcomes (5, 13–16). However, studies comparing IVL with other balloon-based plaque modification techniques are scarce.

To our knowledge, this is the first study carried out in a real-world cohort that investigates the procedural success and outcomes of lesion preparation using MB or IVL in patients with calcified coronary lesions.

## Methods

### Patient selection and study design

**Figure 1:**
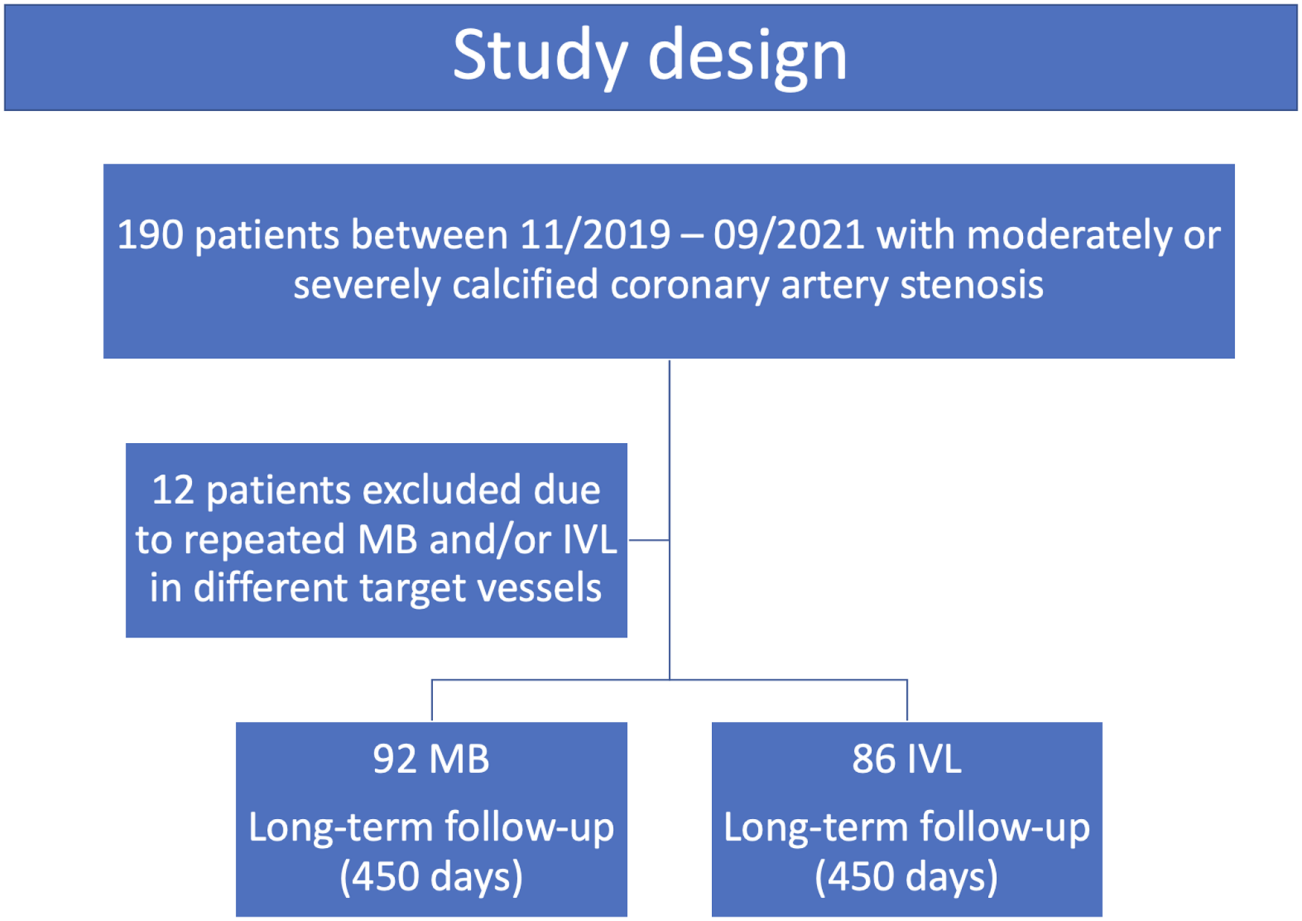
Study flow chart. Patients with moderately to severely calcified coronary lesions were included consecutively between 11/2019 and 09/2021. IVL, intravascular lithotripsy; MB, modified balloon.

This prospective, observational, two-center study based on an all-comers registry included consecutive patients with moderate or severe coronary calcification treated with MB or IVL. The patient screening took place at two German centers (Heart Centre Trier and German Heart Centre Munich) between November 2019 and September 2021. Overall, 190 patients were included in the study (Figure 1). All of these patients had moderately or severely calcified *de novo* stenosis or ISR >50% in one target vessel with symptoms of angina pectoris and/or a positive diagnosis of non-invasive ischemia. Twelve patients were excluded because of MB and/or IVL therapy in more than one target vessel.

Coronary angiography and intervention were performed by interventional cardiologists according to current international standards (17). Patients in whom plaque modification was performed using a cutting or scoring balloon were allocated to the MB group (*n* = 92) (WOLVERINE™ Cutting Balloon™, Boston Scientific, Marlborough, MA, USA; NSE Alpha™, B. Braun Melsungen, Germany). The size of the final MB used was chosen in a 1:1 ratio according to the vessel size. Patients treated by IVL for lesion preparation were assigned to the IVL group (*n* = 86). The shockwave C2 (Shockwave Medical Inc., Santa Clara, CA, USA) balloon-based coronary system was used for IVL, utilizing a 1:1 ratio of IVL balloon to planned stent diameter as recommended by the manufacturer. Use of either balloon-based technique for lesion preparation was at the discretion of the interventional cardiologist. Similarly, further interventional therapy, i.e., drug-eluting stent or drug-eluting balloon (DEB) in some cases of ISR as well as postdilatation after MB or IVL treatment, was also left to the discretion of the operator.

Calcified lesions were angiographically graded into moderate or severe (16). In brief, moderate calcification was defined as radiopacities noted only during the cardiac cycle before contrast dye injection. Severe calcification was defined as radiopacities seen without cardiac motion before the contrast medium was injected (1, 16, 18). Either intravascular ultrasound or optical coherence tomography was recommended and was used at the operator's discretion.

Informed consent was obtained from each patient and in cases of ISR, the patients were informed by the operator about the off-label use before using IVL. The study was approved by ethics committee of the Rhineland-Palatinate chamber of physicians.

### Quantitative coronary angiography

**Figure 2:**
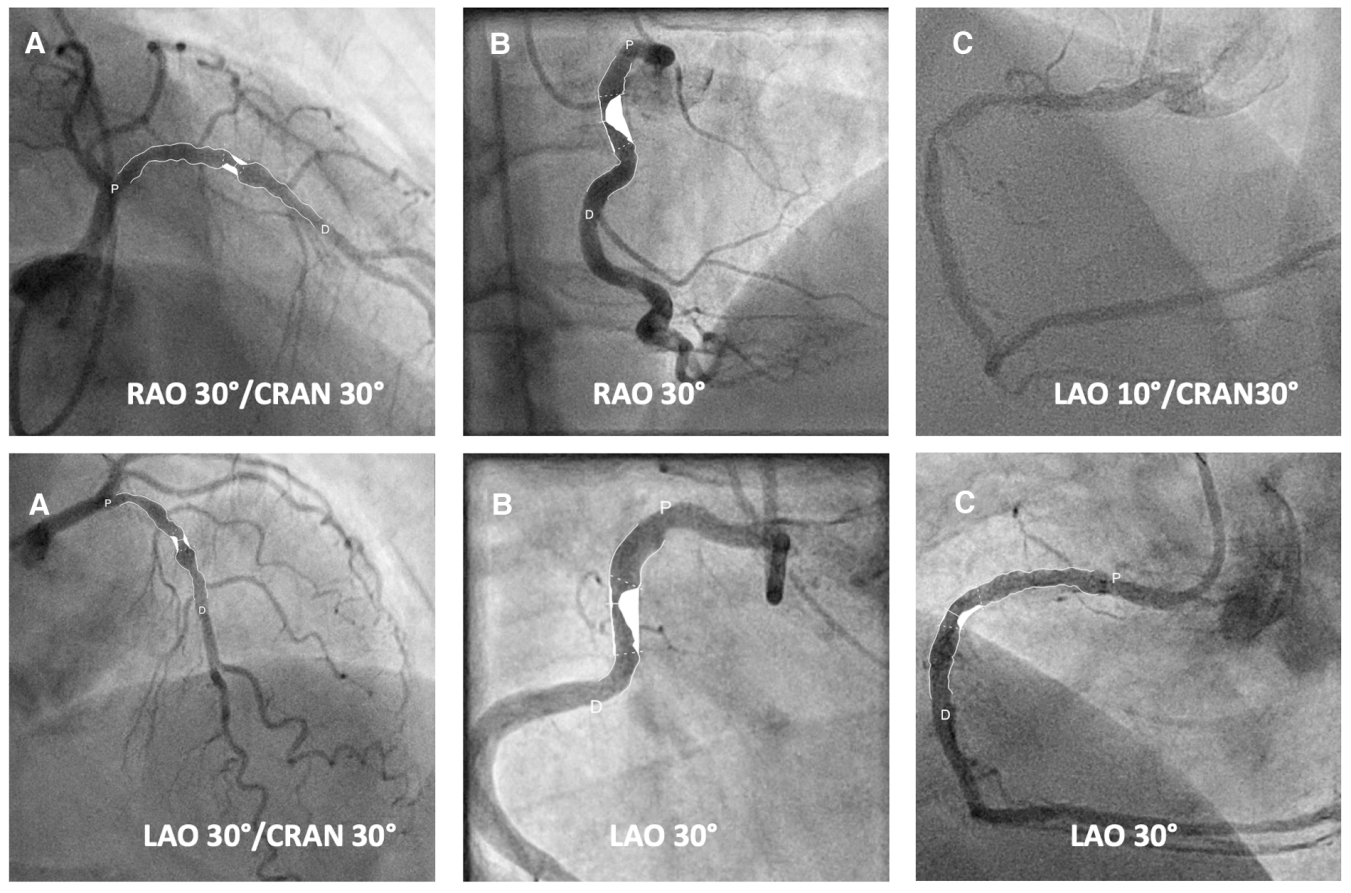
Quantitative coronary angiography. (**A**) Imaging with contrast fluid. Severely calcified concentric stenosis of the left anterior descending coronary artery in two orthogonal projections stenosis (P, proximal; D, distal). (**B**) Imaging with contrast fluid. Severely calcified eccentric stenosis of the right coronary artery in two orthogonal projections stenosis (P, proximal; D, distal). (**C**) Severely calcified stenosis of the proximal right coronary artery and Quantitative coronary angiography of the post-procedural angiographic result showing a 33% residual stenosis (P, proximal; D, distal).

Offline quantitative coronary angiography (QCA) was performed and evaluated by an interventional cardiologist blinded to the procedure groups. Baseline QCA was performed before MB, IVL, or predilatation (Figures 2A,B). Categorization into eccentric or concentric stenosis was done in two orthogonal projections. The angiographic results were scored as treatment success (residual stenosis <20%) or failure (residual stenosis >20%) by the blinded QCA operator (Figure 2). For the primary endpoint analysis measurements were performed using the same single, worst-view projection.

### Follow-up

During their hospital stay, patients were monitored for the occurrence of any adverse events. The following in-hospital adverse events were recorded: cardiopulmonary resuscitation during PCI, coronary artery dissection, peri-interventional acute myocardial infarction (AMI), coronary artery perforation, thrombus formation, stent-thrombosis during hospital stay.

The long-term follow-up assessment was conducted by telephone interview or, in cases of rehospitalization, in person. One patient was lost to follow-up. For all other patients, a follow-up period of at least 450 days was available.

### Endpoints

The primary endpoint was strategy success, which was defined as successful DES/DEB delivery and expansion with less than 20% residual stenosis of the target lesion and TIMI (thrombolysis in myocardial infarction) 3 flow (16). Secondary endpoints were in-hospital major adverse cardiovascular events (MACE), defined as cardiac death, AMI, and target vessel revascularization (TVR) (including target lesion failure) as proposed by American Heart Association and the Academic Research Consortium-2 in the fourth universal definition for myocardial infarction associated with PCI, as well as MACE during the long-term follow-up (19).

### Statistical analysis

The present study design of a non-randomized observational study bears the risk that the assignment of patients to the groups (MB vs. IVL) is not random (probability = 50%), but depends on known and unknown patient characteristics. Propensity score matching was waived because it would significantly reduce the sample size. An alternative approach is to weight each patient's exposure using inverse probability of treatment weighting (IPTW); use of this method in the present study did not lead to a reduction in the sample size. If a random assignment of subjects to groups (intervention group, control group) is not possible, it cannot be excluded that a difference of the subjects already existed before the intervention. In the context of propensity score matching, treatment and control elements with similar values are paired or discarded via the propensity score. This is an attempt to reduce selection bias. A possible reduction of the sample size can have a negative effect. Against this background, the estimation of the bias by IPTW is particularly useful in combination with regression analyses. Based on propensity scores, this parameter is determined as a measure for the bias within the sample. For example, if a logistic regression reveals a significant difference between the intervention group and the control group, but this difference is dependent on other factors (unrelated to group), then when the IPTW measure is used, these effects should no longer become significant.

In the logistic regression analysis performed, there was an effect of the origin of stenosis (*de novo* vs. ISR; *p* < 0.001) on the patient group. No significant effect of other variables on the patient group was found. Due to the difference described above, the origin of stenosis was used as a weighting variable. Overall, the data showed a significant and relevant bias regarding patient group caused by the variable origin of stenosis; this bias could be corrected by applying IPTW (*p* < 0.001 vs. *p* = 0.999).

Data for categorical variables are expressed as values and percentages. For continuous variables, data are reported as the mean ± SD or as the median with interquartile range (IQR), where appropriate.

Multiple logistic regression, including weighting, was used to evaluate the categorical data on the achievement of the secondary endpoint. For model optimization, variable selection was performed in each case in the sense of backward selection. In addition to the *p*-values, an estimate value was obtained for each predictor, interpretable as a log odds ratio (OR). The proportion of variance explained by the models is indicated by an appropriate coefficient of determination (R-squared).

The observations on the achievement of the primary endpoints were evaluated by survival analyses with determination of univariate Cox proportional hazard ratio and Kaplan-Meier curve. Weighting was also included here.

Depending on the scale level, individual variables were tested with the t-test or chi-squared test. The prerequisites for the application of test procedures (variance homogeneity, distribution assumptions, frequencies, etc.) in the determination of *p*-values were taken into account and tested nonparametrically, if necessary. The analysis was performed with R 4.2.2 (R Foundation for Statistical Computing, Vienna, Austria).

## Results

### Baseline and angiographic characteristics and procedural data

A total of *n* = 178 patients were included in the final study cohort. After weighting using IPTW, there were no meaningful differences in the distribution of any baseline clinical characteristics between the IVL and MB groups (Table 1). In patients treated with IVL, one-vessel CAD was less frequent (*p* = 0.001), and two-vessel CAD was more frequent (*p* = 0.001). The presence of acute coronary syndrome at the time of admission was less frequent in the IVL group (*p* = 0.023).

**Table 1:** Baseline characteristics and clinical presentation (*n* = 178).

Variable	MB (*n* = 92)	IVL (*n* = 86)	*p*-value (unadjusted)	*p*-value (weighted)
Baseline characteristics				
Male sex	76 (82.6)	71 (82.6)	0.993	0.501
Age, years	71 (±10.2)	74.2 (±9.6)	0.997	0.994
Hypertension	86 (93.5)	83 (96.5)	0.363	0.426
Current smoker	16 (17.4)	9 (10.5)	0.188	0.203
Former smoker	37 (40.2)	32 (37.2)	0.681	0.522
Diabetes	31 (33.7)	37 (43.0)	0.201	0.257
BMI, kg/m^2^	28.5 (±4.7)	27.9 (±4.9)	0.997	0.991
Hyperlipoproteinemia	89 (96.7)	76 (88.4)	**0** **.** **044**	0.47
Family history of CAD	26 (28.3)	16 (18.6)	0.132	0.613
Chronic kidney disease	19 (20.7)	21 (24.4)	0.548	0.178
End-stage renal disease	0 (0)	2 (2.3)	0.995	0.995
CAD and clinical presentation				
CAD 1	14 (15.2)	7 (8.1)	0.149	**0** **.** **001**
CAD 2	20 (21.7)	25 (29.1)	0.262	**0** **.** **011**
CAD 3	58 (63.0)	54 (62.8)	0.972	0.648
Acute coronary syndrome	27 (29.3)	17 (19.8)	0.141	**0** **.** **023**
NYHA I, baseline	31 (33.7)	24 (27.9)	0.404	0.589
NYHA IV, baseline	5 (5.4)	7 (8.1)	0.475	0.109
CCS I, baseline	28 (30.4)	25 (29.1)	0.842	0.224
CCS IV, baseline	18 (19.6)	11 (12.8)	0.224	0.067
LVEF, %	54.4 (±9.4)	54.6 (±12.1)	0.997	0.997

Data are shown as *n* (%) for dichotomous and as mean (±standard deviation) for continuous variables. Weighted *p*-values are reported after inverse probability treatment weighting (IPTW). Bold *p*-values indicate statistical significance. BMI, body mass index; CAD, coronary artery disease, 1-, 2-, or 3-vessel; CCS, Canadian Cardiovascular Society grading of angina pectoris; IVL, intravascular lithotripsy; LVEF, left ventricular ejection fraction; MB, modified balloon; NYHA, New York Heart Association functional classification.

Lesion characteristics are shown in Table 2. ISR lesions were less frequent in patients treated with IVL (*p* = 0.001). Therefore, ISR was used as a weighting variable for comparison. After IPTW, no significant differences were observed (*p* = 0.999). Severe calcification (*p* = 0.001) as well as tortuosity of coronary arteries (*p* = 0.001) were more frequent in the IVL group.

**Table 2:** Lesion and procedural characteristics (*n* = 178).

Variable	MB (*n* = 92)	IVL (*n* = 86)	*p*-value (unadjusted)	*p*-value (weighted)
Target vessel				
LM	4 (4.3)	5 (5.8)	0.657	0.129
LAD	35 (38.0)	27 (31.4)	0.353	**0** **.** **027**
LCX	23 (25)	12 (14)	0.067	**0** **.** **002**
RCA	30 (32.6)	42 (48.8)	**0** **.** **028**	**0** **.** **001**
Lesion characteristics				
In-stent restenosis^a^	81 (88.0)	36 (41.9)	**0** **.** **001**	0.999
*De novo* lesions^a^	11 (12.0)	50 (58.1)	**0** **.** **001**	0.999
Degree of stenosis, %	84 (±9.7)	86.3 (±10.4)	0.997	0.997
Lesion length: <10 mm	49 (53.3)	35 (40.7)	0.094	0.399
Lesion length: 10–20 mm	31 (33.7)	32 (37.2)	0.624	0.805
Lesion length: >20 mm	12 (13.0)	19 (22.1)	0.115	0.433
Concentric stenosis	73 (79.3)	64 (74.4)	0.436	0.715
Calcified portion of lesion, mm	8.6 (±3.8)	9.6 (±3.6)	0.366	0.212
Severe calcification	28 (30.4)	72 (83.7)	**0** **.** **001**	**0** **.** **001**
Tortuosity	21 (22.8)	34 (39.5)	**0** **.** **017**	**0** **.** **001**
Bifurcation	23 (25.0)	26 (30.2)	0.435	0.358
Procedural characteristics				
Procedure time, min	57.6 (±33)	78.7 (±32.2)	0.997	0.997
x-ray dose, Gy^a^cm^2^	44.3 (±33.5)	59.9 (±39.9)	0.997	0.994
Contrast amount, ml	204.2 (±66.1)	222.2 (±86.2)	0.997	0.997
Pre-dilatation	63 (68.5)	73 (84.9)	**0** **.** **011**	**0** **.** **029**
Pre-dilatation pressure, atm	19.7 (±3.7)	22.6 (±8)	0.487	0.351
Post-dilatation	85 (92.4)	77 (89.5)	0.507	**0** **.** **043**
Post-dilatation pressure, atm	19.5 (±6.4)	22.3 (±7.5)	0.584	0.704
DES implantation	50 (54.3)	83 (96.5)	**0** **.** **001**	**0** **.** **001**
DEB application	41 (44.6)	2 (2.3)	**0** **.** **001**	**0** **.** **001**
Length of DES/DEB, mm	21.8 (±7.5)	27.6 (±12.6)	0.085	**0** **.** **001**
Diameter of DES/DEB, mm	3.3 (±0.5)	3.6 (±0.6)	0.996	0.995

Data are shown as *n* (%) for dichotomous and as mean (standard deviation) for continuous variables. Weighted *p*-values are reported after inverse probability treatment weighting (IPTW). Bold *p*-values indicate statistical significance. DEB, drug-eluting balloon; DES, drug-eluting stent; LM, left main artery; LAD, left anterior descending artery; LCX, left circumflex artery; IVL, intravascular lithotripsy; MB, modified balloon; RCA, right coronary artery.

^a^ Weighting variable.

Procedural data are shown in Table 2. Predilatation of the target lesion was more frequent (*p* = 0.029), and post-dilation was less frequent (*p* = 0.043) in the IVL group. DES implantation was more common in patients treated with IVL (*p* = 0.001). The cumulative drug-eluting stent or -balloon length was longer in the IVL group (*p* = 0.001). No other significant differences were observed.

### Procedural and in-hospital events

No procedural complications occurred in the MB group. Intravascular thrombus formation was observed in two patients of the IVL group. One of these patients fulfilled the criteria for a type 4 myocardial infarction. One dissection of the target vessel was observed in the IVL group during the procedure. Furthermore, we observed cardiopulmonary resuscitation for two cycles due to ventricular tachycardia in one patient in the IVL group (PCI of the right coronary artery). No vascular perforation or acute stent thrombosis occurred in either group. No slow flow or no reflow events were observed in either group. There were no TVR events or cardiac deaths during the in-hospital period.

### Primary efficacy endpoint

**Figure 3:**
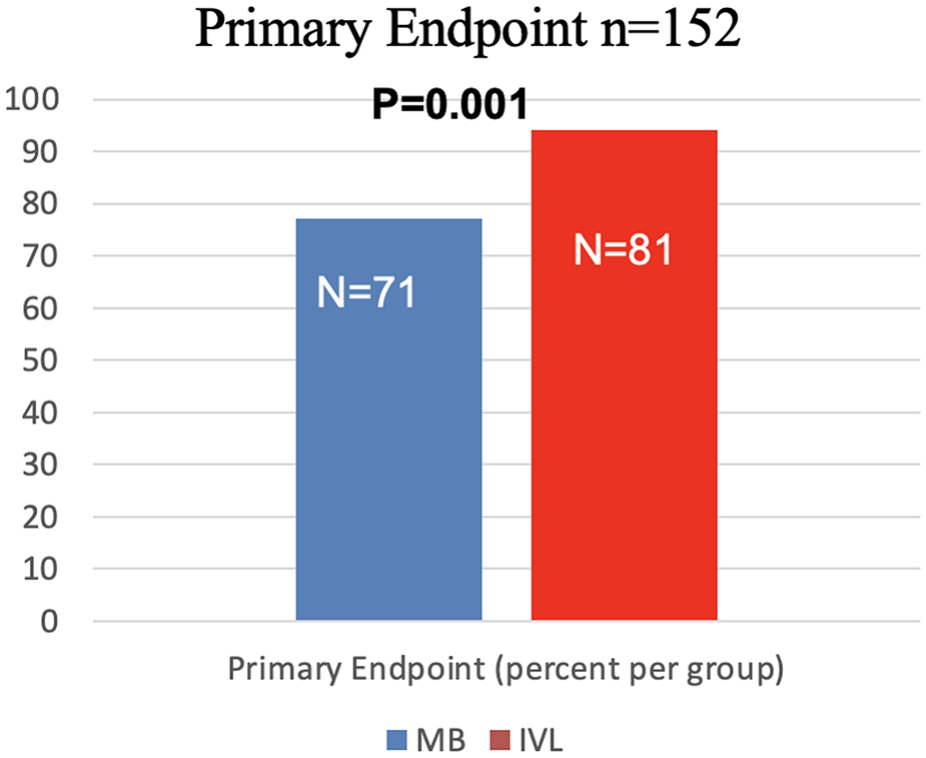
Strategy success for MB and IVL. Strategy success was the primary endpoint with success rates of 94.2% in IVL group, and 77.2% in MB group (*p* = 0.001).

The primary endpoint was reached in 152 patients (Figure 3: Overall: 85.4%; IVL: 94.2% vs. MB 77.2%; *p* = 0.001). Only 5 (5.8%) patients in the IVL group had residual stenosis vs. 21 (22.8%) in the MB group (*p* = 0.001) in QCA.

Weighted multivariable regression analysis revealed that IVL had a positive effect on reaching the primary endpoint (estimate 3.202; standard error 0.661; *p* = 0.001) (Table 3). Further, patients with concentric calcification (*p* = 0.017), chronic kidney disease (*p* = 0.034), and one-vessel CAD disease (*p* = 0.016) also had a higher probability of reaching the primary endpoint. Overall, however, this probability was lower than that due to the variable IVL. In addition, patients who were former smokers (*p* = 0.023) or had end-stage renal disease (*p* = 0.003) or severe calcification (*p* = 0.001) had a lower probability of achieving the primary endpoint. No other baseline or procedural characteristics had a significant effect on the primary endpoint in the multivariable regression analysis.

**Table 3:** Weighted multivariate regression analysis—effect on primary endpoint.

Variable	Estimate (OR)	Standard error (95% CI)	*p*-value (weighted)
Intervention group			
IVL	3.202 (24.58)	0.661 (7.4–101.86)	**0** **.** **001**
Baseline and procedural characteristics			
Former smoker	−1.406 (0.25)	0.617 (0.07–0.79)	**0** **.** **023**
Chronic kidney disease	1.702 (5.48)	0.801 (1.30–32.60)	**0** **.** **034**
End-stage renal disease	−5.78 (0.003)	1.973 (0.001–0.177)	**0** **.** **003**
Severe calcification	−2.548 (0.08)	0.619 (0.02–0.24)	**0** **.** **001**
Concentric calcification	1.245 (3.47)	0.523 (1.26–9.97)	**0** **.** **017**
CAD 1	1.672 (5.32)	0.692 (1.51–23.24)	**0** **.** **016**
Cumulative DEB/DES length, mm	0.063 (1.07)	0.032 (1.00–1.14)	0.053
Diameter of DES/DEB, mm	0.839 (2.31)	0.521 (0.83–6.55)	0.107

Data are shown as estimate and standard error as well as odds ratio (OR) and 95% confidence interval (95% CI) in the brackets for better comparison. Bold *p*-values indicate statistical significance. CAD 1, coronary artery disease in 1 vessel; IVL, intravascular lithotripsy; DEB, drug-eluting balloon; DES, drug-eluting stent.

In an unweighted subgroup analysis of patients with ISR, the overall procedural success rate was 84.6%. Patients in the IVL group had less residual stenosis (*n* = 1 [2.8%] vs. *n* = 17 [21.0%]; *p* = 0.012). Multivariable regression analysis revealed that IVL also had a positive effect on reaching the primary endpoint (estimate 2.857; OR 17.4; standard error 1.166; *p* = 0.014) in patients with ISR.

### Secondary endpoints—long-term follow-up

With the exception of one patient from the IVL group, long-term follow-up data were available for all other patients. During the long-term follow-up period 5 patients in the IVL group and 3 patients in the MB group died (2.8% vs. 1.7% *p* = 0.129). Patients for whom the cause of death was unknown were classified as cardiac death. Weighted univariate Cox proportional hazard analysis showed no difference between treatment modalities on cardiovascular mortality [hazard ratio (HR) 7.457; 95% confidence interval (95% CI) 0.824–67.477; *p* = 0.074]. Patients with unstable angina (CCS IV) at time of the index procedure had the highest probability of cardiovascular death (HR 7.136; 95% CI 1.248–40.802; *p* = 0.027). Further, the degree of stenosis (HR 1.119; 95% CI 1.047–1.196; *p* = 0.001) and LVEF at discharge after the index procedure (HR 0.936; 95% CI 0.882–0.993; *p* = 0.017) also had an effect on the mortality in the hazard analysis.

No significant difference was found in the long-term rate of AMI (IVL *n* = 3 (1.7%) vs. MB *n* = 5 (2.8%); *p* = 0.399; IVL HR 2.73; 95% CI 0.4–17.0; *p* = 0.281). In the weighted univariate Cox proportional hazard analysis, the degree of stenosis had a significant impact on the occurrence of an AMI during the follow-up (HR 1.102; 95% CI 1.044–1.164; *p* = 0.001). The rate of target lesion failure/revascularization (IVL *n* = 10 [5.6%] vs. MB *n* = 16 [9%]; *p* = 0.186; IVL HR 0.78; 95% CI 0.277–2.166; *p* = 0.626) was also without significant difference between the groups. Weighted univariate Cox proportional hazard analysis revealed that DES implantation had a positive effect on the rate of TVR (HR 0.346; 95% CI 0.125–0.959; *p* = 0.041). In addition, predilatation pressure was associated with a higher risk of TVR (HR 0.097; 95% CI 1.034–1.175; *p* = 0.003). No other differences were observed.

**Figure 4:**
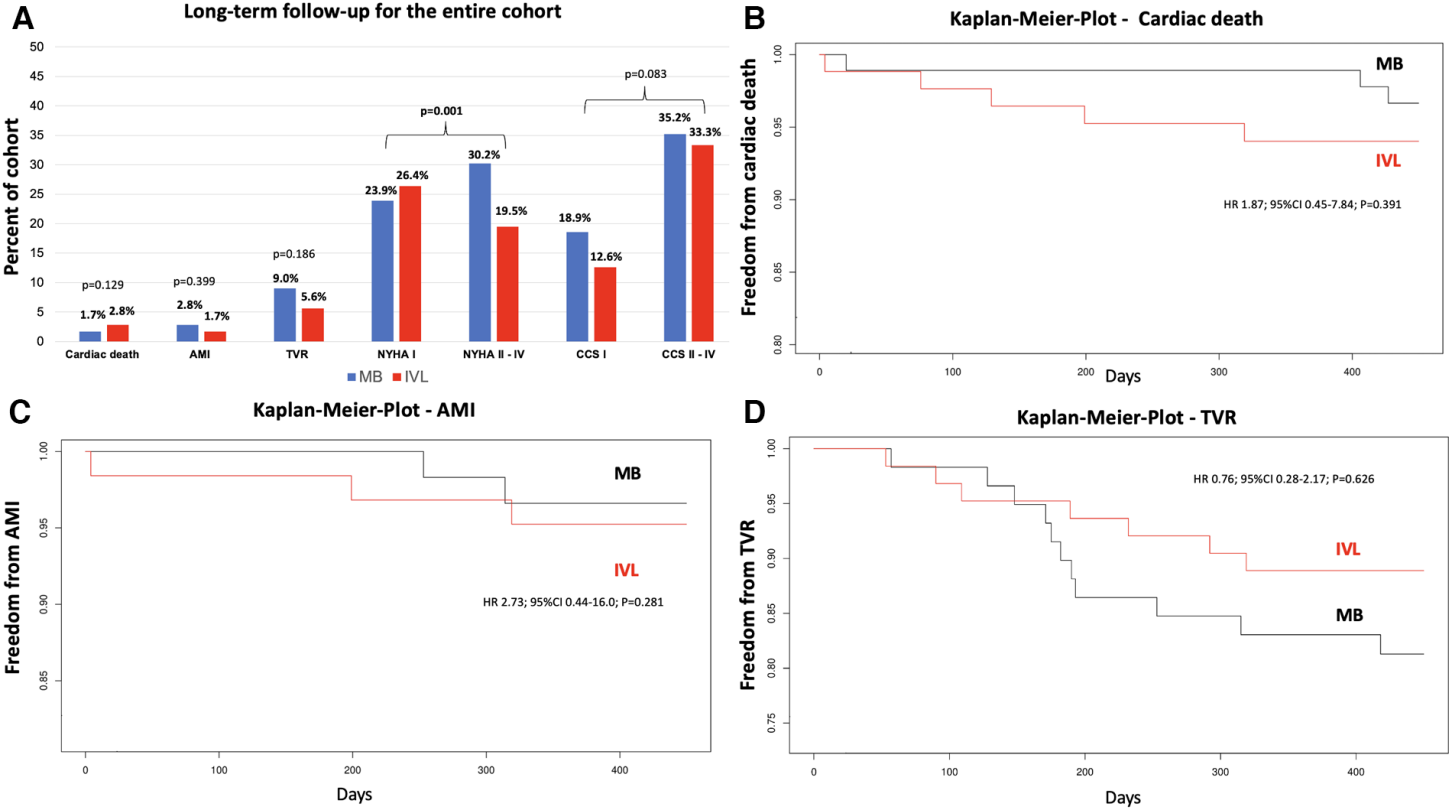
Long-term follow-up and kaplan-meier analysis of secondary endpoints for patients treated by MB or IVL. Long-term follow-up (**A**): values represent percent of cohort treated with either MB or IVL. Plots demonstrates freedom from cardiac death (**B**) acute myocardial infarction (**C**) and target vessel revascularization (**D**) during long-term follow-up. IVL, intravascular lithotripsy; MB, modified balloon; AMI, acute myocardial infarction; TVR, target vessel revascularization; CCS, Canadian Cardiovascular Society grading of angina pectoris; NYHA, New York Heart Association functional classification; HR, hazard ration; 95% CI, 95% confidence interval. *p*-values are reported after inverse probability treatment weighting. Bold *p*-values indicate statistical significance.

Kaplan-Meier analysis of freedom from cardiac death (Figure 4B), freedom from AMI (Figure 4C), and freedom from TVR (Figure 4D) revealed no significant differences between the groups.

NYHA and CCS grades could be reported by 73 (5 deceased patients; 7 frailty patients, 1 patient no data available) patients in the IVL group and 86 patients in the MB group (3 deceased patients; 3 frailty patients). Patients in the IVL group reported less limitation of physical activity due to dyspnea compared with those in the MB group (NYHA class I: IVL 26.4% (*n* = 42) vs. MB 23.9% (*n* = 38); NYHA class II–IV: IVL *n* = 31 (19.5%) vs. MB *n* = 48 (30.2%); *p* = 0.001) during the follow-up (Figure 4A). There was no difference between patients with CCS I class (IVL *n* = 20 (12.6%) vs. MB *n* = 30 (18.9%) and those with CCS II–IV class (IVL *n* = 53 (33.3%) vs. MB *n* = 56 (35.2%); *p* = 0.083) between the groups. However, the singular proportion of CCS IV class patients was higher in the MB group (IVL *n* = 2 (1.3%) vs. MB *n* = 11 (6.9%); *p* = 0.025).

## Discussion

To the best of our knowledge, this is the first study to compare IVL with MB angioplasty for lesion preparation in patients with moderate or severe coronary calcification in an all-comers population. Severe lesion calcification leads to stent underexpansion and has been reported as a predictor of worse outcome in patients with coronary artery calcification (5–7, 20). Studies, particularly randomized trials, that compare the performance of devices in calcified lesions are scarce (11, 21, 22). The reported angiographic success rates vary widely and depend on the chosen strategy, which may include preparation of the lesion before stent implantation by super high-pressure balloon, MB, IVL, or rotational atherectomy devices; success rates between 78% and 99% have been reported (5, 16, 21, 23–26). In our cohort we observed an angiographic success rate of 85% overall. One of our main findings is that patients treated with IVL for lesion preparation had significantly less residual stenosis compared with patients treated with MB.

### Procedural success rates

Strategy success was observed in 94% of the patients in our cohort treated with IVL, which is higher than that of previous studies (5, 13–16, 23, 24, 27). A pooled analysis of all patients enrolled in the Disrupt CAD I–IV trials showed an overall success rate of 92.4%. However, in the Disrupt CAD trials procedural success was defined as residual stenosis less than 30% and patients with ISR were excluded (5). In contrast, Aksoy and colleagues demonstrated a success rate of 78.2% overall and 82.5% in an IVL study cohort that included patients with ISR (16, 23). Higher success rates in the present study compared with the studies cited might be due to patient selection. In these studies, the proportion of severely calcified coronary lesions in the IVL group tended to be higher (82.5%–97.0%) than in our cohort (84%) (5, 16, 23). This was also shown in the multivariable regression analysis of the primary endpoint. Here, severe coronary calcification led to a lower probability of reaching the primary endpoint. A procedural success rate of 99% was reported in a multicenter, observational study; however, in this registry 11% of the patients underwent additional rotational atherectomy and the rate of ISR was lower than in our cohort (26).

In the MB group strategy failure was observed in 23% of the cases. This rate is slightly higher than in other studies, where rates of 18.5%–19% have been described (21, 28, 29). In contrast, the recently published randomized ISAR-CALC trial reported residual stenosis in just 2.7% of cases after MB (11). However, in this study, residual stenosis was defined as >30% and complementary rotational atherectomy was allowed. Further, patients with aorto-ostial stenosis as well as patients with ISR were excluded. Therefore, these data are not comparable with ours (11). The largest trial investigating the use of MB, the Cutting Balloon Global Randomized Trial, randomized a total of 1,238 patients to either MB or semi-compliant balloon dilatation (8). However, this study was performed more than 20 years ago, and DES implantation was considered a bailout. Also, the percentage of patients with residual stenosis >20% was not reported (8). The authors reported an overall residual stenosis of 29% ± 14% after MB (8). Data from the randomized COPS trial showed that the use of a MB (cutting balloon) leads to a significant improvement in minimal stent area compared to a non-compliant balloon (12). However, the primary endpoint differs with our study, so it is not completely comparable with our study. The slightly higher proportion of strategy failure in patients treated with MB in our cohort could be due to the higher rates of ISR, which is often due to inadequate lesion preparation where heavy calcification has prevented complete stent expansion. This was not investigated in these studies.

We attribute the significantly lower rate of residual stenosis in the IVL group than in the MB group to the different mechanism of action of the two techniques. This was also confirmed by the weighted multivariable regression analysis: patients treated with IVL had the highest probability of reaching the primary endpoint. Recent studies have demonstrated that IVL has high procedural success and few complications in patients with coronary artery calcification (5, 13–15, 26). While MB results in a controlled incision of the calcified lesion, IVL can reach the entire circumference of the vessels by converting sound waves into mechanical energy. This is effective in disrupting superficial and deeply embedded calcifications (5, 8, 10, 11, 13–15, 21, 28). In this context, weighted multivariable regression analysis also showed that concentric calcification leads to a higher probability of achieving procedural success.

### Follow-up analyses

At long-term follow-up, there were no significant differences in the secondary endpoints. The largest and longest meta-analysis to date, evaluating the impact of target lesion calcification on clinical outcomes after DES implantation and including more than 6,200 patients, demonstrated that severe calcification is associated with a 44% increased 5-year risk in cardiac death, a 23% increased risk in target vessel AMI, and a 21% increased risk in TVR compared with noncalcified lesions (30). However, due to the novelty of the IVL technique, data regarding long-term follow-up are scarce. In the present study the rates of cardiac death (2.8%), AMI (1.7%), and TVR (5.6%) were low in patients treated with IVL during follow-up. A recent, real-world, multicenter European study also showed low rates of cardiac death (5%), AMI (3%) and TVR (6%) in 273 patients treated with IVL during a median follow-up period of 687 days (26). These data are comparable to ours for a somewhat shorter follow-up period of 450 days. The 1-year results from the Disrupt CAD III study also demonstrated low rates of cardiac death (1.1%), AMI (10.5%), and ischemia-driven TVR (6.0%) (31). In particular, the rate of AMI was higher than in our real-world cohort. We attribute this to the study design, as the Disrupt CAD III was a prospective, single-arm approval study designed to assess the safety and effectiveness of IVL (14).

The 2-year outcome results of the randomized PREPARE-CALC revealed higher rates of TVR (TVR and target lesion failure) in patients treated with a cutting or scoring balloon compared with our findings (20% vs. 9%, respectively) (22). The rates of AMI were comparable (5% vs. 4%). However, because of differently defined secondary endpoints, follow-up duration, and exclusion of patients with an ISR, our data cannot be strictly compared with these data.

Overall, both groups showed a low rate of cardiac death, AMI and TVR although the angiographic outcome was significantly better in the IVL group. From the point of view of cost-benefit analysis, this is problematic, as MB is more cost-effective than IVL. Therefore, in this context, there will be a further follow-up in 3–5 years to proof whether there are significant effects in the secondary endpoint over a longer observation period.

Another finding from our registry is the significant difference in the intensity of dyspnea due to physical activity during follow-up. Patients in the IVL group were less symptomatic than patients in the MB group (NYHA I 26.4% vs. 23.9%). There are no comparable data in the literature. We attribute the higher rate of dyspnea-free patients to the higher success rate in terms of the angiographic outcomes. More residual stenosis may account for more residual symptoms. In this context, a prospective multicenter pilot trial including 144 patients with CAD revealed that PCI effectively reduced the prevalence of dyspnea, demonstrated by a decline from 73% before PCI to 54% afterwards (32).

### Limitations

The present study was non-randomized and was performed in a limited number of patients. Propensity score matching was waived in order to maintain the sample size; IPTW was applied as an alternative approach. Randomized trials, which can overcome the selection bias inherent in all-comers registries like ours, are urgently needed to compare different lesion preparation methods with IVL. However, observational data help in designing endpoints in larger randomized trials, e.g., the inclusion of quality-of-life endpoints given the significant differences in symptoms between IVL and MB patients noted in the present study. A registry cannot control individual, operator-based decisions. Important steps during lesion preparation, including pre- and postdilatation, applied pressure, and choice of DES or DEB, were left to the operator's discretion, which may have influenced the results. Furthermore, systematic angiography during follow-up was not available. Due to the low rate of intravascular imaging (13%) and the use of two different imaging modalities, no analysis was performed here. Lesion characterization was exclusively based on angiographic characteristics mainly due to the lack of established and comparable scores for lesion calcification. However, this reflects the real-world situation of our registry. Systematic collection of cardiac biomarker data before and after the procedure was not mandatory, and collection was performed at different time points.

## Conclusion

In our all-comers cohort, lesion preparation with IVL results in a significantly lower rate of residual stenosis than MB angioplasty. Long-term follow-up showed low rates of cardiac death, TVR, and AMI overall. Based on these results, larger randomized trials are needed to compare safety and efficacy of the different lesion preparation devices currently available for the treatment of moderately to heavily calcified coronary stenoses.

## Data Availability

The raw data supporting the conclusions of this article will be made available by the authors, without undue reservation.
